# Implications of early diagnosis of autosomal dominant polycystic kidney disease: A *post hoc* analysis of the TEMPO 3:4 trial

**DOI:** 10.1038/s41598-020-61303-9

**Published:** 2020-03-09

**Authors:** Peter Janssens, François Jouret, Bert Bammens, Max C. Liebau, Franz Schaefer, Ann Dandurand, Ronald D. Perrone, Roman-Ulrich Müller, Christina S. Pao, Djalila Mekahli

**Affiliations:** 1 PKD Research Group, Laboratory of Pediatrics, University Hospitals Leuven, Leuven, Belgium; 2 Department of Nephrology, University Hospitals Brussels, Brussels, Belgium; 3 Division of Nephrology, University of Liège Hospital (ULiège CHU), Liège, Belgium; 4 Groupe Interdisciplinaire de Génoprotéomique Appliquée (GIGA), Cardiovascular Sciences, ULiège, Liège Belgium; 5 Department of Microbiology & Immunology, KU Leuven, Leuven Belgium; 6 Department of Nephrology, Dialysis and Renal Transplantation, University Hospitals Leuven, Leuven, Belgium; 7 Department of Pediatrics and Center for Molecular Medicine, University of Cologne, Faculty of Medicine and University Hospital of Cologne, Cologne, Germany; 8 Department II of Internal Medicine and Center for Molecular Medicine Cologne, University of Cologne, Faculty of Medicine and University Hospital of Cologne, Cologne, Germany; 9 Cologne Excellence Cluster on Cellular Stress Responses in Ageing-Associated Diseases (CECAD) and Systems Biology of Ageing Cologne (Sybacol), University of Cologne, Faculty of Medicine and University Hospital of Cologne, Cologne, Germany; 10 Division of Pediatric Nephrology, University Children’s Hospital Heidelberg, Heidelberg, Germany; 11 Otsuka Pharmaceutical Development & Commercialization Inc., Princeton, USA; 12 Division of Nephrology, Tufts Medical Center and Tufts University School of Medicine, Boston, USA; 13 Department of Pediatric Nephrology, University Hospitals Leuven, Leuven, Belgium

**Keywords:** Paediatric research, Polycystic kidney disease

## Abstract

It is unknown whether early diagnosis of autosomal dominant polycystic kidney disease (ADPKD) can enable earlier management and improve outcomes. We conducted a *post hoc* analysis of data from the TEMPO 3:4 trial. Subjects were stratified by ADPKD diagnosis at age ≤18 (childhood diagnosis [CD]) or>18 (adulthood diagnosis [AD]). Groups were compared for baseline characteristics and total kidney volume (TKV) growth and estimated glomerular filtration rate (eGFR) decline over 3 years. 294 CD and 1148 AD subjects were analyzed. At inclusion, CD subjects were younger (mean age 34.2 versus 39.8 years; *p* < 0.0001) and had better eGFR (mean ± SD 87.4 ± 23.9 versus 80.1 ± 20.7 mL/min/1.73 m^2^; *p* < 0.0001), while CD had more severe Mayo risk classification (*p* < 0.0001) and more *PKD1* mutations (*p* = 0.003). No statistical differences were found in TKV or eGFR change. At study end, placebo-treated CD subjects had better eGFR than projected by a prediction equation (mean difference ±SD for observed versus predicted eGFR: 2.18 ± 10.7 mL/min/1.73 m^2^; *p* = 0.0475). However, these results are not confirmed when excluding stage 1 CKD. Whether CD subjects, despite their risk profile, have a slower disease course than predicted remains inconclusive. Future studies are needed to confirm that early diagnosis and management can alter the disease course of ADPKD.

## Introduction

Autosomal dominant polycystic kidney disease (ADPKD) is an inherited disorder characterized by progressively enlarging cystic kidneys. The disease process starts prenatally but often remains pauci-symptomatic and with little or no detectable effect on kidney function during the first decades of life. As cyst burden increases, patients experience a gradual loss of functioning nephrons and are likely to experience burdensome symptoms such as kidney pain or abdominal distension. Eventually, the parenchymal destruction caused by cyst formation and growth leads to end stage renal disease (ESRD) in more than half of patients by the age of 60^1^. Since cysts formed early in life contribute disproportionally to the final total cyst volume^2^, it might be argued that the best chance for preserving long-term renal function would be to start treatment at the first opportunity. At the same time, early diagnosis and follow-up in asymptomatic at-risk children remains controversial. Potential benefits of treatment need to be balanced against factors such as psychological stress, legal/financial implications resulting from the knowledge of having a progressive disease, and potential adverse events of pharmacotherapy in a slowly progressing disease^3^.

Medications for ADPKD used to be limited to antihypertensive treatment with the goal of improving renal and cardiovascular outcomes^4^. The renin-angiotensin-aldosterone system (RAAS) is upregulated in APDKD and might contribute to cyst growth and kidney function decline. The use of RAAS-inhibitory antihypertensive agents in an early-stage population was shown to achieve a low blood pressure target and modestly inhibit the growth of total kidney volume (TKV)^5^. Recently, prospective studies in adult patients at risk for rapid ADPKD progression have demonstrated that vasopressin V2 receptor antagonism, which directly targets pathways implicated in ADPKD cystogenesis, significantly slows TKV increase and estimated glomerular filtration rate (eGFR) decline^6–9^. This has led to an entirely new treatment paradigm for adults with ADPKD. However, no disease-modifying therapeutic options are currently available for ADPKD patients younger than 18 years, and the potential benefits of early interventions like antihypertensive treatment and RAAS inhibition remain debated. Still, studies of disease-modifying treatments, such as an ongoing phase IIIb trial focusing on the safety and efficacy of tolvaptan in ADPKD-affected children aged 4 to 14 years (NCT02442674), may change the treatment paradigm for pediatric ADPKD patients.

At this point, data on the natural history of and prognostic indicators for childhood ADPKD are both largely lacking and urgently needed^10–12^. The Tolvaptan Efficacy and Safety in Management of ADPKD and Its Outcomes (TEMPO) 3:4 trial is one of the largest studies conducted in the field (N = 1445)^6^. The data from TEMPO 3:4 include detailed medical histories and 3 years of prospective follow up. Given that 20% of the subjects in this cohort were diagnosed with ADPKD during childhood, the TEMPO 3:4 data represent a unique opportunity to evaluate the effect of early diagnosis on long-term renal outcomes. We evaluated the hypothesis that earlier diagnosis, and therefore potentially earlier initiation of renoprotective therapy, might have benefits over later diagnosis in terms of TKV and eGFR.

## Materials and Methods

All methods were carried out in accordance with relevant guidelines and regulations. The institutional review board or ethics committee at each site approved the protocol; written informed consent was obtained from all participants. A full list of the TEMPO 3:4 investigators and trial sites is given in a Supplementary Appendix. The design of the TEMPO 3:4 study has been described previously^6,13^. In brief, this multicenter, randomized, double-blind, placebo-controlled, parallel-group trial enrolled a population of ADPKD-affected subjects (ages 18–50 years) with relatively preserved eGFR but a high likelihood of rapid disease progression. Key inclusion criteria were creatinine clearance ≥60 mL/min, estimated by the Cockcroft-Gault formula, and TKV ≥ 750 mL. Participants were randomized in a 2:1 ratio to 3 years of treatment with tolvaptan or matching placebo, titrated to twice daily doses of 45/15 mg, 60/30 mg, or 90/30 mg, at the highest level tolerable to the subject.

Data collected during the trial included ADPKD medical history based on subject recollection and, where available, medical records (including the age of diagnosis and the initial symptom); vital signs; laboratory parameters (e.g., hematology, serum chemistry, urinalysis); and ADPKD-related outcomes such as kidney pain, worsening hypertension, and worsening albuminuria. Standardized MRI scans of the kidneys were obtained at baseline and at months 12, 24, and 36. Equations from the Chronic Kidney Disease Epidemiology Collaboration (CKD-EPI) that are adjusted for ethnic group were used to estimate GFR^14,15^.

Subjects were stratified by age at ADPKD diagnosis ≤18 years (childhood diagnosis [CD]) and >18 years (adulthood diagnosis [AD]) and compared for baseline characteristics at study entry and for rates of TKV growth and eGFR decline during the 3-year study follow-up period. CD diagnosed subjects were considered symptomatic patients if the reason for diagnosis was high blood pressure, hematuria, urinary tract infection or kidney pain. The remaining subjects were considered asymptomatic at diagnosis. Statistical comparisons of changes in TKV and eGFR were derived using linear mixed models, in which the intercept had the fixed effect and the slope had both fixed and random effects (for change in TKV) or in which both intercept and slope had fixed and random effects (for change in eGFR).

Observed eGFR was compared to predicted eGFR for each subject based on the prediction model developed by Irazabal and colleagues^16^. Observed eGFR was the eGFR (CKD-EPI) value at last visit on treatment, and predicted eGFR was the value calculated based on the eGFR (CKD-EPI) value at end of titration/week 3, years from end of titration/week 3 to last visit on treatment, sex, age, and Mayo classification. The prediction equation is 21.18-0.23*year + [−1.26(if female)−0.26*age+0.90*eGFR+0.58(if class B)−1.14(if class C)−1.93(if class D)−6.26(if class E)] + [0.19(if female)−0.02*age+0.001*eGFR-1.33(if class B)−2.63(if class C)−3.48(if class D)−4.78(if class E)]*year^16^. A paired T-test was used to compare differences between observed and predicted eGFR.

We performed two sensitivity analyses, the first a linear mixed model with change in eGFR as outcome, adjusted for baseline differences (including AD versus CD) and their interactions with time. Second, we applied a recently developed mixed polynomial model to predict eGFR change over time^17^. The prediction model developed by Irazabal *et al*. assumes a linear relation between age and eGFR slope, whereas kidney function in ADPKD is relatively stable at young age and declines more rapidly later. The mixed polynomial model accounts for this nonlinearity.

## Results

### Baseline characteristics

The study population of TEMPO 3:4 included 294 (20%) CD subjects and 1148 (80%) AD subjects at enrollment. The distribution of ages at diagnosis in the CD and AD groups is shown in Supplementary Fig. 1. Subject baseline characteristics are shown in Table 1.

**Table 1:** Clinical characteristics of childhood diagnosis and adulthood diagnosis subjects at enrollment in TEMPO 3:4.

Parameter	CD			AD			*p*-value: Total CD vs Total AD
	Tolvaptan (n = 196)	Placebo (n = 98)	Total (n = 294)	Tolvaptan (n = 762)	Placebo (n = 386)	Total (n = 1148)	
Male sex, n (%)	99 (51)	48 (49)	147 (50)	395 (52)	203 (53)	598 (52)	NS
RAASi use, n (%)	134 (68)	65 (66)	199 (68)	554 (73)	285 (74)	839 (73)	NS
Hypertension, n (%)	158 (81)	80 (82)	238 (81)	627 (82)	325 (84)	952 (83)	NS
CKD stage, n (%)*							0.0015^†^
CKD I	81 (41)	47 (48)	128 (44)	248 (33)	126 (33)	374 (33)	
CKD II	83 (42)	42 (43)	125 (43)	381 (50)	182 (47)	563 (49)	
CKD III	32 (16)	8 (8)	40 (14)	131 (17)	77 (20)	208 (18)	
Mayo risk class, n (%)*							<0.0001^†^
1B	5 (2.6)	4 (4.1)	9 (3.1)	68 (8.9)	27 (7.0)	95 (8.3)	
1 C	44 (22.4)	27 (27.6)	71 (24.1)	295 (38.7)	164 (42.5)	459 (40.0)	
1D	75 (38.3)	31 (31.6)	106 (36.1)	257 (33.7)	130 (33.7)	387 (33.7)	
1E	68 (34.7)	32 (32.6)	100 (34.0)	111 (14.6)	50 (13.0)	161 (14.0)	
2	2 (1.0)	3 (3.1)	5 (1.7)	26 (3.4)	14 (3.6)	40 (3.5)	
Mean age at inclusion (SD)	34.3 (8.0)	34.0 (8.3)	34.2 (8.1)	39.6 (6.4)	40.1 (6.2)	39.8 (6.4)	<0.0001
Mean eGFR (SD)	85.6 (24.0)	91.0 (23.5)	87.4 (23.9)	80.2 (20.1)	79.9 (22.0)	80.1 (20.7)	<0.0001
Median TKV (IQR)	1533 (1092, 2161)	1451 (1048, 2058)	1508 (1075, 2153)	1441 (1072, 2019)	1473 (1052, 1965)	1453 (1068,1990)	0.2545

Age in years; eGFR in mL/min/1.73 m^2^, TKV in mL.

*Includes subjects for whom the data were available. Percentages may not add up to 100. ^†^*p*-value is for the distribution of CKD stages/Mayo risk classes for CD versus AD.

AD, adulthood diagnosis; CD, childhood diagnosis; CKD, chronic kidney disease; IQR, interquartile range; NS, not significant.

CD subjects were significantly younger at time of study enrollment than AD subjects and had significantly better eGFR. The distribution of CKD stages was, accordingly, milder in the CD subjects; the AD group had higher proportions of subjects in CKD stages 2 and 3. Although the CD subjects were younger, they exhibited similar TKV to AD subjects, most likely due to the study inclusion criteria. Younger mean age and comparable TKV at study inclusion in the CD group relative to the AD group corresponded to a significantly worse Mayo risk class distribution in the CD group, with approximately 70% of the CD subjects and fewer than 50% of the AD subjects in classes 1D-1E. Data on genetic diagnosis in the two groups are shown in Table 2. At inclusion, proteinuria was present in 24.0% of the AD group and 24.8% in the CD group. The causes of diagnosis in the CD group are shown in Table 3. The cause of diagnosis in most subjects (70%) was categorized as “Others,” given that this information was not required to be collected.

**Table 2:** Genetic analysis.

	CD (N = 294) n (%)	AD (N = 1143) n (%)	*p*-value (two sided)
Genetic analysis	160 (54.4)	607 (53.1)	NS
No mutation found	6 (3.8)	13 (2.1)	NS
*PKD1*	147 (91.9)	508 (83.7)	0.003
Truncating	106 (66.2)	360 (59.3)	NS
Non-Truncating	44 (27.5)	155 (25.5)	NS
*PKD2*	7 (4.4)	86 (14.2)	<0.001

AD, adulthood diagnosis; CD, childhood diagnosis; NS, not significant.

**Table 3:** Reasons for diagnosis in the childhood diagnosis cohort.

Cause	N = 294
Hypertension, n (%)	30 (10.2)
Hematuria, n **(**%)	14 (4.8)
Urinary tract infection, n (%)	21 (7.1)
Kidney pain, n (%)	26 (8.8)
Unknown, n (%)	12 (4.1)
Others, n (%)	207 (70.4)

Rates of change in TKV and eGFR during the study.

**Figure 1:**
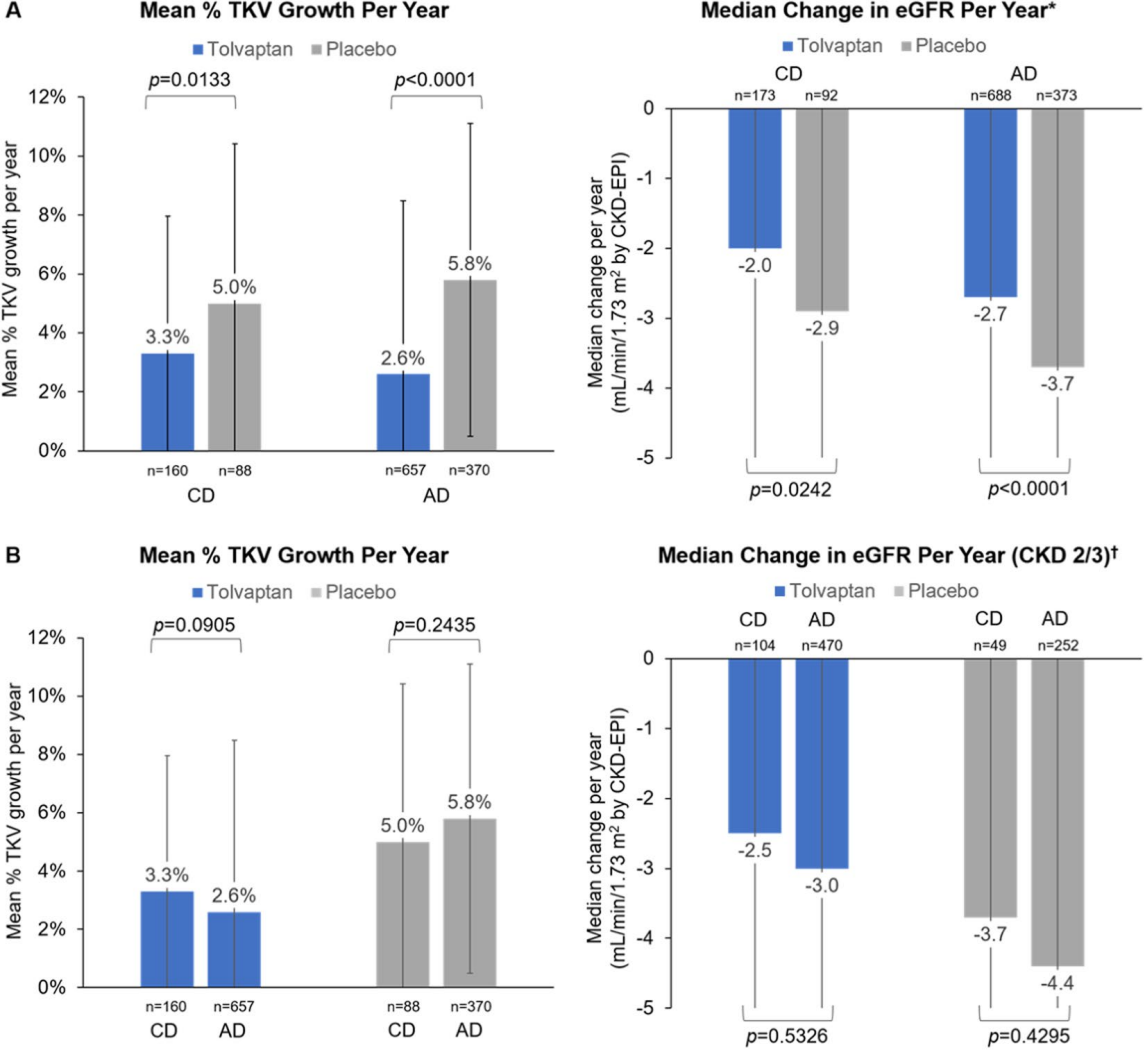
Annual TKV growth and change in eGFR: (**A**) for tolvaptan versus placebo within the childhood diagnosis and adulthood diagnosis subgroups; (**B**) for childhood diagnosis versus adulthood diagnosis subjects within the tolvaptan and placebo study arms (intention-to-treat subjects). *Excludes observations deemed unreliable by investigators: 19 outliers of 4759 data points in the placebo group and 16 outliers of 8564 data points in the tolvaptan group. ^†^Subjects in CKD stages 2 or 3 (CKD stage 1 excluded). Excludes observations deemed unreliable by investigators. Error bars are standard deviation. AD, adulthood diagnosis; CD, childhood diagnosis; CKD-EPI, Chronic Kidney Disease Epidemiology Collaboration; eGFR, estimated glomerular filtration rate; TKV, total kidney volume.

Consistent with the findings for the overall study population in TEMPO 3:4, tolvaptan was associated with significant decreases in the rates of TKV growth and eGFR decline in both the CD and AD subgroups (Fig. 1A). In a subsequent analysis, CD and AD subgroups within each study treatment arm were compared (Fig. 1B). To better discern intergroup differences, only subjects in CKD stages 2 or 3 were included in this eGFR comparison, as eGFR is more likely to decline at later CKD stages. There were no significant differences in rates of TKV growth or eGFR decline. In the placebo-treated arm, TKV growth and eGFR decline were milder in the CD subgroup versus the AD subgroup, but the differences were not significant. There were also no significant differences in eGFR decline between the CD and AD subgroups within each treatment arm when all subjects (CKD stages 1–3) were included in the comparison (data not shown).

To minimize potential bias from the use of arbitrary age categories (≤18 years and >18 years), a linear mixed models was performed using age at diagnosis as a continuous variable. Results did not show significant effects of age at diagnosis on the model (Supplementary Tables 1–3). A sensitivity analysis evaluating baseline characteristics and their interactions with time, however, supported the association of age at diagnosis with eGFR outcome, yielding a significant age at diagnosis x time interaction (*p* = 0.0015) (Supplementary Table 4).

An additional analysis assessed for a possible impact of cause of ADPKD diagnosis among the CD subjects, given that outcomes might differ based on whether a patient is diagnosed early while asymptomatic (for example, screening because of familial history) or because of symptoms associated with a more severe phenotype (hypertension, hematuria, urinary tract infection, or kidney pain). The subanalysis was conducted within the limitation that the reasons for diagnosis were unavailable for most subjects. As would be expected, hypertension and use of RAAS inhibitors were more common at study baseline in subjects diagnosed due to symptoms than in asymptomatic subjects (Supplementary Table 5). Tolvaptan exerted significant effects on TKV increase and eGFR decline versus placebo in asymptomatic CD subjects but not in subjects diagnosed due to symptoms, possibly because of the small size of the analysis population (Supplementary Tables 6 and 7).

**Figure 2:**
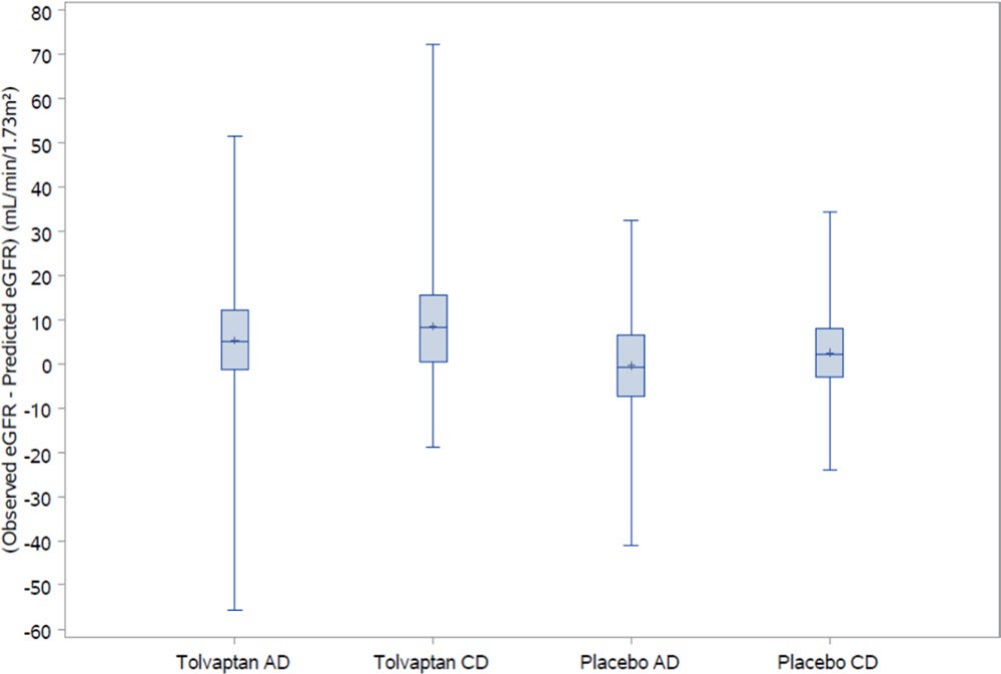
Observed–predicted eGFR (mL/min/1.73 m^2^) within the treatment period in the adulthood diagnosis and childhood diagnosis subgroups. AD, adulthood diagnosis; CD, childhood diagnosis; eGFR, estimated glomerular filtration rate.

Next, the difference between observed and predicted eGFR values was plotted for CD and AD subjects (CKD stages 1–3) in each of the tolvaptan and placebo arms over the study follow-up period (Fig. 2). As might be expected, the observed eGFR was significantly higher than the predicted eGFR in tolvaptan-treated subjects (mean difference ± SD of 6.86 ± 11.1 mL/min/1.73 m^2^ [*p* < 0.0001] for CD and 4.40 ± 10.5 mL/min/1.73 m^2^ [*p* < 0.0001] for AD). Interestingly, in placebo-treated subjects, the predicted eGFR was not different from the observed eGFR in AD subjects (mean difference ± SD of 0.07 ± 10.5 mL/min/1.73 m^2^ [*p* = 0.8995]). However, in the CD subjects, the observed eGFR at the end of follow up was significantly higher than the predicted eGFR (mean difference ± SD of 2.18 ± 10.7 mL/min/1.73 m^2^ [*p* = 0.0475]).

To account for differences between the AD and CD populations, additional analyses controlling for subject age at study inclusion, Mayo risk class, and CKD stage were conducted. Among a subgroup of AD and CD subjects matched by Mayo risk class and age at study inclusion, the difference between observed and predicted eGFR was still significant in tolvaptan-treated subjects, whereas there was no significant difference in either AD or CD placebo-treated subjects (Supplementary Table 8). A subgroup analysis that excluded subjects at CKD stage 1 at baseline (i.e., included only subjects at CKD stages 2–4) yielded significant differences between observed and predicted eGFR in tolvaptan-treated AD and CD subjects, placebo-treated AD subjects, but not placebo-treated CD subjects (Supplementary Table 9). An analysis of CD subjects by reason for diagnosis (subjects diagnosed due to symptoms and other subjects) showed a significant difference between observed and predicted eGFR for tolvaptan-treated subjects but not placebo-treated subjects, possibly due to the small analysis population (Supplementary Table 10). Finally, a sensitivity analysis based on the mixed polynomial prediction model developed by Yu *et al*.^17^ found no significant differences in observed versus predicted eGFR in either tolvaptan- or placebo-treated subjects defined by age at diagnosis (Supplementary Table 11).

## Discussion

We report the first *post hoc* analysis of long-term renal outcome data from a large ADPKD cohort diagnosed during childhood. The placebo group in particular represents a unique opportunity to study the natural history of ADPKD diagnosed in children. Twenty percent of the subjects studied in the TEMPO 3:4 trial were diagnosed with ADPKD at or before the age of 18 years. At the time of inclusion, the CD group was significantly younger, with a better eGFR but a similar TKV relative to the AD group. Consequently, the CD group had a significantly worse Mayo risk class distribution at enrollment. Significantly more *PKD1* mutations were found in the CD group; inversely, more *PKD2* mutations were found in the AD group. The younger age at inclusion of the CD group (34 years compared to 40 years, *p* < 0.0001) would be expected, given that the groups were defined based on their age at diagnosis.

Several arguments could be made that the CD group would be at risk for a more rapidly progressive form of ADPKD. While the higher eGFR in the CD subjects versus AD subjects could be explained by the younger average age of the CD group, the presence of compensatory hyperfiltration, a factor associated with renal progression, could be postulated^18^. It is assumed that in ADPKD, kidney function remains normal for several decades because of hyperfiltration by remaining healthy nephrons. Yet, hyperfiltration remains a poorly defined entity^19^, and a recent study in young, early-stage ADPKD patients did not demonstrate hyperfiltration, defined as a decreased difference in kidney function reserve capacity after a dopamine infusion^20^. The present study does not address the potential occurrence of compensatory glomerular hypertrophy.

CD subjects had significantly more severe Mayo class, as approximately 70% of the CD subjects and fewer than 50% of the AD subjects were classes 1D-1E. A worse Mayo classification implies that a more progressive disease is expected. An increase in TKV is widely accepted as being the dominant feature of ADPKD progression, as TKV is inherently linked to the pathogenesis of PKD and represents a primary event rather than a secondary consequence of disease-causing mutations^21^. Indeed, it was recently demonstrated that the proportion of the total effect of gene type that is mediated by Mayo class is 0.73, supporting the notion that *PKD* mutations cause loss of GFR predominantly by affecting kidney size^17^. A htTKV ≥600 mL/m at baseline predicts the development of stage 3 CKD within 8 years^22^, and patients with higher rates of TKV growth at baseline have an increased frequency of ESRD after 10 years^16^. The predicting renal outcomes in ADPKD (PROPKD) model is unsuitable for assessing the risk of progression in the CD group, which had a mean age of 34 years, given that PROPKD scoring cannot be applied in patients aged <35 unless they have already experienced ADPKD-related clinical events^23^. More data on the progression of ADPKD and potential predictive risk markers early in life are needed.

Consistent with the more severe Mayo imaging classification, more subjects in the CD group had a *PKD1* mutation. It is well known that patients with *PKD1* mutations in general have a more severe renal phenotype than patients with *PKD2* mutations, reaching ESRD almost 20 years earlier^24^. It is thus possible that patients with *PKD1* mutations are diagnosed earlier, e.g., in case of severe family history. Indeed, the TEMPO 3:4 trial included patients with a high likelihood of rapid progression and a selection bias for *PKD1* mutations is suspected. Further, an early and rapidly progressive ADPKD phenotype might be in part explained by additional (hypomorph) mutations in other ciliopathy genes^25^. Although these data are currently not available for the TEMPO 3:4 cohort, whole-genome sequencing might yield additional prognostic information in the CD population^26^.

No difference in hypertension and RAAS inhibition was noted between CD and AD subjects at enrollment. However, as the CD subjects were 5 years younger, this might signify an earlier diagnosis and treatment of hypertension in the CD group. No information on potential counseling that AD subjects had received, such as the avoidance of kidney injury or increased water-drinking behavior, was recorded in the study.

In the untreated (placebo) arm, CD subjects experienced somewhat lower rates of change in TKV and eGFR than AD subjects, although the differences were not significant. Additional analyses using age at diagnosis as a continuous variable did not show significant effects on outcomes. Despite the aforementioned risk factors for rapid progression, placebo treated CD subjects had a significantly better eGFR (mean difference 2.18 mL/min/1.73 m^2^ [*p* = 0.0475]) at the end of follow-up than predicted. By contrast, the prediction model was accurate in AD subjects (mean difference 0.07 mL/min/1.73 m^2^ [*p* = 0.8995]). The significant difference between observed and predicted eGFR in the CD group must be interpreted within the context of multiple comparisons and the possibility of a chance finding. Although the Mayo prediction equation is validated and commonly used as a clinical tool, a sensitivity analysis based on the mixed polynomial prediction model recently developed by Yu *et al*.^17^ found no significant differences in observed versus predicted eGFR in either tolvaptan- or placebo-treated subjects defined by age at diagnosis.

Several comments need to be made on the Mayo prediction model. First, the validity of the prediction equation is not documented for young patients, as it based on a vast majority of adult patients. With a mean age at inclusion of CD patients of 32.2 years this should not be an issue. More important, the trajectory of eGFR loss is often nonlinear and initially relatively stable when kidney function is still preserved. This renders the estimate less accurate in patients with CKD stage 1^16^. As the CD group contains more CKD stage 1 patients, this could have played a role in the difference between the observed and predicted GFR. Indeed, when subjects with CKD stage I were excluded, the difference between the observed and predicted GFR disappeared. Moreover, while the tolvaptan-treated CD group expectedly performed better than predicted, the difference (6.86 mL/min/1.73 m^2^ in 3 years) was quite large. While in placebo-treated subjects in the AD group the rate of eGFR loss was on average what was predicted (0.07 mL/min/1.73 m^2^), this is surprising because the difference between placebo and tolvaptan treatment was actually 0.98 mL/min/1.73 m^2^ per year in the TEMPO 3:4 trial. These findings suggest that application of the Mayo prediction model has limitations that should be taken into account in the interpretation of the present study.

Although a lack of power should be acknowledged, additional reason for caution is provided by subgroup analyses that accounted for differences between CD and AD subjects in age at study inclusion, Mayo risk class, and CKD stage, and which found no significant differences between predicted and observed eGFR in placebo-treated CD subjects. Similarly, in subgroups of placebo-treated CD subjects defined by reason for ADPKD diagnosis, there were no significant differences between predicted and observed eGFR.

Despite a more severe risk profile of the CD subjects, some analysis of the current post hoc study suggested a less rapid disease course of this group than predicted. However, other analyses could not confirm this finding and more studies are therefore needed before conclusions can be drawn. Although the current data do not contradict the attractive hypothesis that early targeting of risk factors for disease progression, such as hypertension, proteinuria and urological complications could improve the natural history of the disease, this needs to be validated in studies with a more adjusted design.

Since CD patients were diagnosed earlier, they could theoretically be targeted early with disease-modifying treatments. However, several limitations for such strategies are to be taken into consideration. Although different prognostic indicators have been identified in adults^16,23^, no stratification risk factors for progression are validated for children^10^. Nevertheless, because parenchymal destruction already occurs under the cover of compensated GFR values^27^, such indicators are required in order to accurately identify patients at risk for rapid progression. Earlier treatment restricted to those at risk for rapid disease progression might improve both cost-effectiveness and the benefit-to-risk ratios of therapies. It is noteworthy that tolvaptan therapy was associated with similarly decreased rates of TKV growth and eGFR decline in both the CD and AD groups. Both the TEMPO 3:4 and the REPRISE studies demonstrated a greater efficacy of tolvaptan in rapidly progressing subjects. Ongoing studies, such as a phase IIIb trial of tolvaptan in ADPKD-affected children aged 4 to 18 years (NCT02442674), might make the need for prognostic assessment even more relevant.

While tolvaptan is the only disease modifying treatment currently available, it’s considerable side effect profile should be taken into account in young patients. Specifically, the increased aquaresis that can be associated with hydroureteronephrosis and bladder enlargement as demonstrated in children with nephrogenic diabetes insipidus warrants caution^28^. Future studies are needed to evaluate disease-modifying therapies in young ADPKD populations and to delineate balanced safety profiles compatible with a long-term treatment.

This study is limited by its *post hoc* nature. Such analyses are nonetheless useful for generating hypotheses for future study. The data reported explore the possibility that earlier diagnosis and initiation of ADPKD management might improve renal function over the long-term. More prospective studies from a young age could refine prognostic tools, including genetic diagnosis, and determine treatment strategies with optimal risk-benefit profiles for the individual patient.

## Supplementary information

Supplementary Information.
