# Supplementary material for: Implications of early diagnosis of autosomal dominant polycystic kidney disease: A post hoc analysis of the TEMPO 3:4 trial
Source: Sci Rep. 2020 Mar 9;10:4294. doi: 10.1038/s41598-020-61303-9 (PMC7062834; doi:10.1038/s41598-020-61303-9)

SUPPLEMENTARY INFORMATION

Implications of early diagnosis of autosomal dominant polycystic kidney disease: A *post hoc* analysis of the TEMPO 3:4 trial

Authors

Peter Janssens, François Jouret, Bert Bammens, Max C. Liebau, Franz Schaefer, Ann Dandurand, Ronald D. Perrone, Roman-Ulrich Müller, Christina S. Pao, Djalila Mekahli

Contents

Appendix: Trial Investigators and Sites

Supplementary Tables

Supplementary Figure

Appendix: Trial Investigators and Sites

Argentina: *Hospital Italiano de Buenos Aires, Gascon, Buenos Aires*; De La Fuente, *Hospital Privado — Centro Médico de Córdoba Naciones Unidas, Córdoba*; *Hospital Universitario Austral, Buenos Aires*; *Hospital Privado — Centro Médico de Córdoba, Córdoba*; *Sanatorio Allende Hipólito, Córdoba*; *Instituto de Nefrología, Buenos Aires*; *Hospital Municipal de Vicente López, Dr. Bernardo, Houssay, Buenos Aires*. ***Australia***: *Royal Adelaide Hospital, Adelaide, SA*; *Melbourne Renal Research Group, Melbourne, VIC*; *Princess Alexandra Hospital, Woolloongabba, QLD*; *Royal Melbourne Hospital Grattan, Melbourne, VIC*; *Royal North Shore Hospital, Sydney, NSW*; *Westmead Hospital, Sydney, NSW*; *Renal Research, Gosford, NSW*; *Queen Elizabeth Hospital, Adelaide, SA; Royal Perth Hospital, Perth, WA; Royal Melbourne Hospital Grattan, Melbourne, VIC.* ***Belgium:*** *UCL-Saint-Luc, Brussels; UZ Gen, Ghent; UZ-VUB Brussels, Brussels.* ***Canada:*** *Royal Victoria Hospital, Montréal, Québec; Hôpital du Sacre-Coeur de Montréal, Montréal, Québec; Queen Elizabeth II Health Science Centre, Nova Scotia.* ***Denmark:*** *Odense Universitets Hospital, Odense; Herlev Amtssygehus, Herlev.* ***France:*** *CHU — Hôpital Nord, Saint-Etienne; CHU — Hôpital Lapeyronie, Montpellier; Hôpital Rangueil, Toulouse; CHU-Hôpital Pellegrin, Bordeaux; Hôpital de la Conception, Marseille; Hôpital Edouard Herriot, Lyon; Hôpital Bichat-Claude Bernard, Paris; Centre Hospitalier Universitaire, Reims Cedex; CHU — Hôpital Clemenceau, Clemenceau.* ***Germany:*** *Nephrologische, Düsseldorf; Universitätsklinikum Carl Gustav Carus, Dresden; Klinik für Nephrologie, Essen; Klinik für Nephrologie, Essen; UH Erlangen/Nuernberg, Nuernberg; Universitätsklinikum Freiburg, Freiburg; Nierenzentrum Heidelberg, Ruprecht-Karls-Universität, Heidelberg.* ***Italy:*** *UO Nefrologia, Livorno; Policlinico, Napoli; Policlinico di Modena, Modena; Università Vita e Salute, Milano; Ospedali Riuniti di Bergamo, Bergamo; IRCCS Fondazione Salvatore Maugeri, Pavi.* ***Japan:*** *Fukushima Medical University Hospital, Fukushima; Tokai University Hospital, Kanagawa; Hamamatsu University School of Medicine, Shizuoka; Kyorin University Hospital, Tokyo; Saitama Medical Center, Saitama; Teikyo University Hospital, Tokyo; The Jikei University Hospital, Tokyo; Kyorin University Hospital; Tokyo, Nippon Medical School Hospital, Tokyo; Osaka University Hospital, Osaka; Osaka University Hospital, Osaka; Osaka City University Hospital, Osaka; Tohoku University Hospital, Miyagi; Kitasato University Hospital, Kanagawa; National Hospital Organization, Chiba; Fukushima Medical University Hospital, Fukushima; Jichi Medical School Hospital, Tochigi; Shuwa General Hospital, Saitama; Hiroshima University Hospital, Hiroshima; Hokkaido University Hospital, Hokkaido; Niigata University Medical and Dental Hospital, Niigata; Chiba University Hospital, Chiba; Chiba University Hospital, Chiba; Hokkaido University Hospital, Hokkaido; Tokyo Women's Medical, Tokyo; Kyorin University Hospital, Tokyo; Ohno Memorial Hospital, Osaka; Tokyo Medical and Dental University, Tokyo; National Hospital Organization, Kyoto; Hokkaido University Hospital, Hokkaido; National Hospital Organization, Kyoto; Fujita Health University Hospital, Aichi; Fujita Health University Hospital, Aichi; Saitama Medical Center, Saitama; oranomon Hospital, Tokyo; Kumamoto University Hospital, Kumamoto; Shuwa General Hospital, Saitama; Kyusyu University Hospital, Fukuoka; Toranomon Hospital Kajigaya, Kangawa; Fukushima Medical University Hospital, Fukushima; Hiroshima University Hospital, Hiroshima; Kitasato University Hospital, Kanagawa; Fujita Health University Hospital, Aichi; Fujita Health University Hospital, Aichi.*

Netherlands: *UMCG Groningen, Gronigen; VU Medisch Centrum, Amsterdam.* ***Poland****: Samodzielny Publiczny Szpital Kliniczny, Szczecin. Szpital Praski, Samodzielny, Warszawa; Oddział Nefrologiczny Stacja Dializ, Ciechanow; Akademicki Szpital Kliniczny im J Mikulicza, Wroclaw; SPSzK nr 4 w Lublinie, Klinika Nefrologii, Lublin; Międzyleski Szpital Specjalistyczny w Warszawie, Warszawa; SPSzk nr 4 w Lublinie, Lublin; B. Rutkowski, Akademickie Centrum Kliniczne AMG, Gdansk; Klinika Chorób Wewnętrznych I Nefrologii, Warszawa; Szpital Uniwersytecki w Krakowie, Krakow.* ***Romania****: Spitalul Clinic, Iasi, G. Mircescu, Spitalul Clinic de Nefrologie, Bucharest, Institutul Clinic Fundeni, Bucuresti;* ***Russian Federation****: Kemerovo Medical Academy, Kemerovo, Leningrad; Regional Clinical Hospital, St.Petersburg; The Municipal Health Institution, Municipal Clinical Hospital, Novosibirsk; City Mariinskiy Hospital, St. Petersburg; Tomsk Regional Clinical Hospital, Tomsk; City Clinical Hospital, Moscow.* ***UnitedKingdom****: Queen Elizabeth Hospital, Birmingham; Brighton and Sussex University Hospitals, Brighton; Brighton and Sussex University Hospitals, Brighton; Raigmore Hospital, Inverness; King's College Hospital, London; St. George's Hospital Medical School, London; Belfast City Hospital, Belfast; Morriston Hospital, Swansea; Royal Infirmary, Edinburgh; Center for Nephrology, University College Medical School, London; Royal Hallamshire Hospital, Sheffield; UHCW MHS TRUST, Coventry.* ***United States of America****: Nephrology Associates of Westchester, Hawthorne, NY; Northwestern University, the Feinberg School of Medicine, Chicago, IL; Northwest Renal Clinic, Inc., Portland, OR; University Hospitals of Cleveland, Cleveland, OH;The Rogosin Institute, New York, NY; East Carolina University, Greenville, NC; Charleston Nephrology Associates, N. Charleston, SC; Emory University Hospital, Atlanta, GA; University of South Alabama, Mobile, AL; Yale University School of Medicine, New Haven, CT; University of Colorado Health Sciences Center, Aurora, CO; Kidney & Hypertension Center, Cincinnati, OH; University of Pennsylvania, Philadelphia, PA; Nephrology Associates, Nashville, TN; Coastal Nephrology Associates, Port Charlotte, FL; Jacksonville Center for Clinical Research, Jacksonville, FL; Stanford University Medical Center, Stanford, CA; Coastal Clinical Research, Mobile, AL; Apex Research of Riverside, Riverside, CA; Yale University Medical School, New Haven, CT; University of North Carolina, UNC Kidney Center, Chapel Hill, NC; Tufts Medical Center, Boston, MA; Stanford University Medical Center, Stanford, CA; Columbia University Medical Center, New York, NY; Renal Associates of Baton Rouge, Baton Rouge, LA; University of Virginia, Nephrology Clinical Research Center, Charlottesville, VA; Vanderbilt University Medical School, Nashville, TN; Northwest Renal Clinic, Inc., Portland, OR; Beth Israel Deaconness Medical Center, Boston, MA; Mayo Clinic, Rochester, MN; Northwestern University, Chicago, IL; Erie County Medical Center, Buffalo, NY; Johns Hopkins School of Medicine, Baltimore, MD; University of Kansas Medical Center, Kansas City, KS.*

Originally published as a supplementary appendix to: Torres VE, Chapman AB, Devuyst O et al; TEMPO3:4 Trial Investigators. Tolvaptan in patients with autosomal dominant polycystic kidney disease. N Engl J Med 2012; 367: 2407–2418.

Supplementary Tables

Supplementary Table 1. TKV rate of growth (%/year) within the treatment period, age at diagnosis as a continuous variable (intention-to-treat subjects)

		Annualized % Growth Rate					Slope (%)			Treatment Effect (%)				
**Treatment Group**	**N**	**Mean**	**Median**	**SD**	**Min**	**Max**	**Slope**	**Lower**	**Upper**	**Difference**	**Lower**	**Upper**	***p*-value**	***p*-value for Interaction of Age at Diagnosis**
Tolvaptan	819	2.777	2.265	5.659	-23.129	64.270	2.752	2.410	3.094	-2.670	-3.23	-2.12	<0.0001	0.3026
Placebo	458	5.608	5.585	5.330	-20.634	43.948	5.495	4.926	6.067					

Supplementary Table 2. Rate of change in renal function, estimated by Chronic Kidney Disease-Epidemiology Collaboration equation (mL/min/1.73 m^2), within the treatment period, age at diagnosis as a continuous variable (subjects in CKD stages 2–3 only)

		Rate of Change Per Year					Slope (95% CI)			
**Treatment Group**	**N**	**Mean**	**Median**	**SD**	**Min**	**Max**	**Slope**	**Lower**	**Upper**	***p*-value for Interaction of Age at Diagnosis**
Tolvaptan	574	-1.622	-2.927	18.165	-157.01	245.754	-3.013	-3.263	-2.763	0.6603
Placebo	301	-4.346	-4.244	6.187	-81.283	17.903	-4.306	-4.686	-3.926	0.4437

Supplementary Table 3. Rate of change in renal function, estimated by Chronic Kidney Disease-Epidemiology Collaboration equation (mL/min/1.73 m^2), within the treatment period, age at diagnosis as a continuous variable (intention-to-treat subjects)

		Rate of Change Per Year					Slope (95% CI)			Absolute Treatment Effect (95% CI)				
**Treatment Group**	**N**	**Mean**	**Median**	**SD**	**Min**	**Max**	**Slope**	**Lower**	**Upper**	**Difference**	**Lower**	**Upper**	***p*-value**	***p*-value for Interaction of Age at Diagnosis**
Tolvaptan	863	-1.083	-2.511	25.905	-258.56	476.635	-2.270	-2.935	-2.506	0.980	0.600	1.360	<0.0001	0.3947
Placebo	465	-3.735	-3.481	5.757	-81.283	24.585	-3.700	-4.080	-3.321					

Supplementary Table 4. Sensitivity analysis: linear mixed model of eGFR, intention-to-treat subjects

Variable	Linear Mixed Model (N=1381))		
	**Coefficient**	**95% Confidence Interval**	***p*-value**
Intercept	12.0683	(8.5895, 15.5471)	<.0001
Time, yr	-3.5485	(-5.4151, -1.6819)	0.0002
Male	-0.2018	(-0.7584, 0.3548)	0.4773
Age, yr	-0.1671	(-0.2319, -0.1023)	<.0001
Mayo Class A	-2.0210	(-4.3876, 0.3456)	0.0942
Mayo Class B	2.1363	(0.6742, 3.5983)	0.0042
Mayo Class C	1.2829	(0.3815, 2.1843	0.0053
Mayo Class D	0.9099	(0.1048, 1.7150)	0.0268
Baseline eGFR	0.9161	(0.8985, 0.9338)	<.0001
Age at diagnosis ≤18 years	-0.2803	(-0.9755, 0.4149)	0.4293
Male x time	0.0962	(-0.2538, 0.4462)	0.5899
Age x time	-0.0453	(-0.0821, -0.0084)	0.0161
Mayo Class A x time	4.0204	(1.4437, 6.5972)	0.0022
Mayo Class B x time	2.7224	(1.9136, 3.5313)	<.0001
Mayo Class C x time	1.6828	(1.1004, 2.2652)	<.0001
Mayo Class D x time	1.0431	(0.5073, 1.5790)	<.0001
Baseline eGFR x time	0.0157	(0.0065, 0.0249)	0.0008
Age at diagnosis ≤18 years x time	0.7239	(0.2765, 1.1713)	0.0015

Linear mixed model with variables listed above, where intercept and time are random effects.
eGFR, estimated glomerular filtration rate.

Supplementary Table 5. Baseline characteristics of childhood diagnosis subjects by reason for diagnosis

	With Symptoms		Without Symptoms		
**Parameter**	**N**	**Value**	**N**	**Value**	***p*-value for Comparison**
Male, n (%)	93	46 (49)	201	101 (50)	0.9002
RAASi use, n (%)	93	75 (81)	201	124 (62)	0.0012
Hypertension, n (%)	93	82 (88)	201	156 (78)	0.0320
CKD Stage 1, n (%)	92	42 (46)	201	86 (43)	0.6462
CKD Stage 2, n (%)	92	36 (39)	201	89 (44)	0.4083
CKD Stage 3, n (%)	92	14 (15)	201	26 (13)	0.5975
Age (yr), mean (SD)	93	32.76 (8.85)	201	34.84 (7.62)	0.0400
eGFR (CKD-EPI, mL/min/1.73 m^2), mean (SD)	92	88.48 (26.17)	201	86.91 (22.82)	0.6033
TKV (mL), median (interquartile range)	93	1583 (1208,2419)	201	1453 (1067,2004)	0.0410

CKD, chronic kidney disease; CKD-EPI, Chronic Kidney Disease Epidemiology Collaboration equation; eGFR, estimated glomerular filtration rate; RAASi, renin-angiotensin-aldosterone system inhibitor.

Supplementary Table 6. Total kidney volume rate of growth (%/year) within the treatment period in childhood diagnosis subjects by reason for diagnosis

			Annualized % Growth Rate						Slope (%)			Treatment Effect (%)			
**Subgroup**	**Treatment Group**	**N**	**Mean**	**Median**	**SD**	**Min**	**Max**	**Slope**	**Lower**	**Upper**	**Difference**	**Lower**	**Upper**	***p*-value**
CD with symptoms	Tolvaptan	53	4.273	4.140	4.886	-6.504	16.839	4.062	2.774	5.367	-1.794	-4.01	0.370	0.1043
	Placebo	26	6.039	5.513	5.112	-3.484	16.551	5.929	3.677	8.230				
CD without symptoms	Tolvaptan	107	2.863	3.283	4.497	-9.083	13.079	2.940	2.119	3.767	-1.526	-3.05	-0.029	0.0457
	Placebo	62	4.574	5.195	5.531	-8.877	18.327	4.511	2.970	6.075				

CD, childhood diagnosis.

Supplementary Table 7. Rate of change in renal function, estimated by Chronic Kidney Disease-Epidemiology Collaboration equation (mL/min/1.73 m^2), within the treatment period in childhood diagnosis subjects by reason for diagnosis

			Rate of Change Per Year					Slope (95 CI%)			Absolute Treatment Effect (95% CI)				
**Subgroup**	**Treatment Group**	**N**	**Mean**	**Median**	**SD**	**Min**	**Max**	**Slope**	**Lower**	**Upper**	**Absolute Difference**	**Lower**	**Upper**	**Difference (%)**	***p*-value**
CD with symptoms	Tolvaptan	55	-1.043	-2.362	10.310	-15.463	63.709	-2.837	-3.794	-1.880	0.566	-0.871	2.002	16.62	0.4396
	Placebo	27	-3.111	-2.822	5.493	-21.535	14.929	-3.403	-4.839	-1.967					
CD without symptoms	Tolvaptan	118	3.397	-1.811	44.921	-32.102	476.635	-1.994	-2.603	-1.386	1.203	0.173	2.234	37.63	0.0221
	Placebo	65	-3.351	-2.953	5.294	-20.290	17.903	-3.197	-4.228	-2.167					

CD, childhood diagnosis.

Supplementary Table 8. Difference in observed and predicted eGFR within the treatment period in adulthood diagnosis and childhood diagnosis subjects matched by Mayo risk class and age at study inclusion

	Observed eGFR – Predicted eGFR (mL/min/1.73 m^2)						Paired T-test	
	**N**	**Mean**	**Median**	**SD**	**Min**	**Max**	**95% CI of Mean**	***p*-value**
**Tolvaptan-treated**								
CD	180	6.82	6.61	11.22	-18.95	48.03	(5.17. 8.47)	<0.0001
AD	180	5.43	5.36	10.41	-38.14	45.84	(3.90, 6.96)	<0.0001
**Placebo-treated**								
CD	87	1.83	1.72	10.51	-34.62	33.24	(-0.41, 4.07)	0.1080
AD	87	0.63	0.59	9.49	-26.74	27.51	(-1.40, 2.65)	0.5390

AD, adulthood diagnosis; CD, childhood diagnosis.

Supplementary Table 9. Difference in observed and predicted eGFR within the treatment period in adulthood diagnosis and childhood diagnosis subjects, excluding subjects at CKD stage 1 at baseline (i.e., baseline CKD stages 2–4)

	Observed eGFR – Predicted eGFR (mL/min/1.73 m^2)						Paired T-test	
	**N**	**Mean**	**Median**	**SD**	**Min**	**Max**	**95% CI of Mean**	***p*-value**
**Tolvaptan-treated**								
CD	111	4.73	2.59	10.23	-16.59	38.49	(2.81, 6.66)	<0.0001
AD	495	2.99	2.53	9.20	-35.81	29.55	(2.17, 3.80)	<0.0001
**Placebo-treated**								
CD	50	-0.51	0.01	9.03	-23.97	23.65	(-3.08, 2.06)	0.6913
AD	255	-1.91	-1.92	9.59	-32.69	32.39	(-3.09, -0.72)	0.0017

AD, adulthood diagnosis; CD, childhood diagnosis.

Supplementary Table 10. Difference in observed and predicted eGFR within the treatment period in childhood diagnosis subjects by reason for diagnosis

	Observed eGFR – Predicted eGFR (mL/min/1.73 m^2)						Paired T-test	
	**N**	**Mean**	**Median**	**SD**	**Min**	**Max**	**95% CI of Mean**	***p*-value**
**Tolvaptan-treated**								
CD with symptoms	59	7.92	7.45	12.07	-16.59	48.03	(4.77, 11.06)	<0.0001
CD without symptoms	125	6.30	5.15	10.66	-18.95	34.20	(4.41, 8.19)	<0.0001
**Placebo-treated**								
CD with symptoms	28	3.22	5.26	11.74	-34.62	19.19	(-1.33, 7.77)	0.1584
CD without symptoms	69	1.76	1.28	10.28	-22.31	33.24	(-0.72, 4.23)	0.1608

AD, adulthood diagnosis; CD, childhood diagnosis.

Supplementary Table 11. Sensitivity analysis: difference in observed and predicted eGFR within the treatment period in adulthood diagnosis and childhood diagnosis subjects, mixed polynomial eGFR prediction model

	Observed eGFR – Predicted eGFR (mL/min/1.73 m^2)						Paired T-test	
	**N**	**Mean**	**Median**	**SD**	**Min**	**Max**	**95% CI of Mean**	***p*-value**
**Tolvaptan-treated**								
CD	187	-1.51	-0.32	22.54	-47.61	84.61	(-4.76,1.74)	0.3618
AD	727	1.41	1.51	23.46	-74.58	98.12	(-0.29,3.12)	0.1044
**Placebo-treated**								
CD	97	-1.80	0.41	21.66	-58.43	51.61	(-6.17,2.56)	0.4148
AD	378	0.72	1.52	23.88	-67.83	73.98	(-1.70,3.13)	0.5588

The prediction equation is based on Yu et al, *Kidney Int* 2019;95:1253–1261;Supplementary Table S2: [2.40*age-0.04*(age^2)+60.79](if class A), [2.31*age-0.05*(age^2)+72.82](if class B), [0.38*age-0.04*(age^2)+121.58](if class C), [1.74*age-0.07*(age^2)+111.4](if class D), [2.87*age-0.11*(age^2)+98.06](if class E).
AD, adulthood diagnosis; CD, childhood diagnosis, eGFR, estimated glomerular filtration rate.

Supplementary Figure 1. Age at diagnosis in the childhood diagnosis (A) and adulthood diagnosis (B) groups in TEMPO 3:4

A

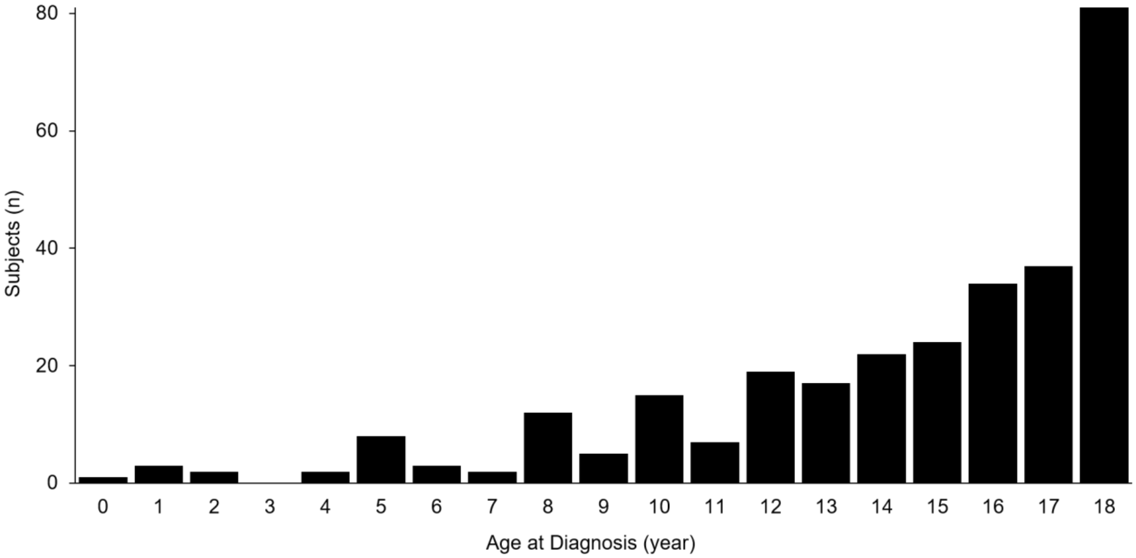

B

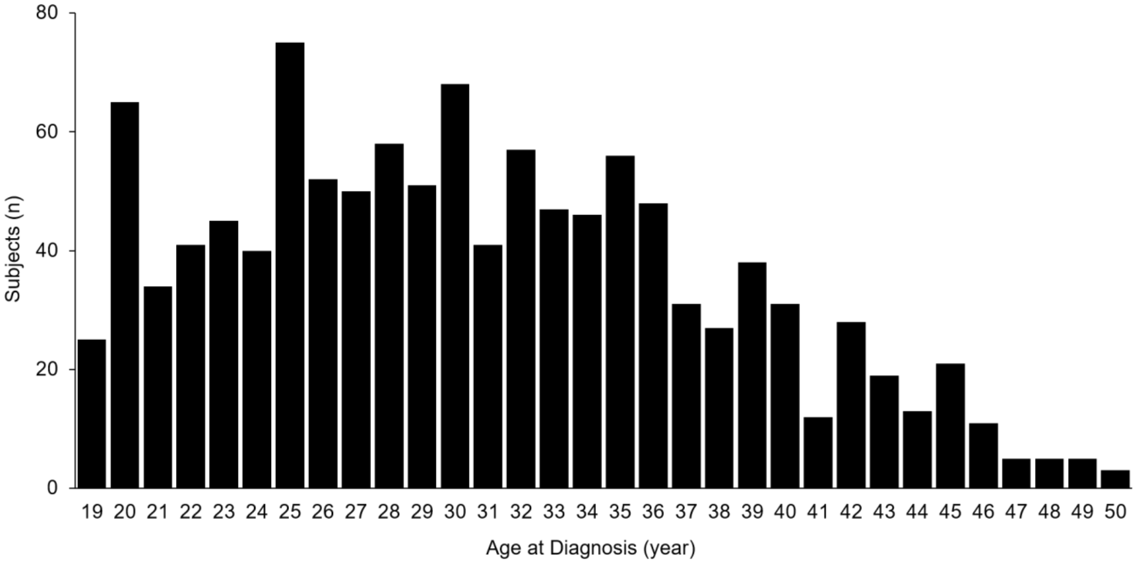

Supplement: Supplementary file 1 — Supplementary Information. [file 41598_2020_61303_MOESM1_ESM.pdf]
